# Immune cell profiling and antibody responses in patients with COVID-19

**DOI:** 10.1186/s12879-021-06278-2

**Published:** 2021-07-05

**Authors:** Mitra Rezaei, Shima Mahmoudi, Esmaeil Mortaz, Majid Marjani

**Affiliations:** 1 Virology Research Center, National Research Institute of Tuberculosis and Lung diseases (NRITLD), Shahid Beheshti University of Medical Sciences, Tehran, Iran; 2 Pediatric Infectious Disease Research Center, Pediatrics Center of Excellence, Children’s Medical Center Hospital, Tehran University of Medical Sciences, Dr. Gharib Street, Keshavarz Boulevard, Tehran, Iran; 3 Clinical Tuberculosis and Epidemiology Research Center, National Research Institute of Tuberculosis and Lung Diseases (NRITLD), Shahid Beheshti University of Medical Sciences, Tehran, Iran

**Keywords:** SARS-CoV-2, COVID-19, Antibody response, Lymphocyte subsets

## Abstract

**Background:**

Although there are a growing number of studies on evaluating lymphocyte subset counts as prognostic factors for COVID-19 disease severity, the lymphocyte subsets’ analyses of both IgM and IgG responders and non-responders during the periods after onset of symptoms, have not been conducted yet. So, this study aimed to evaluate immune cell profiling of COVID-19 patients with and without antibody responses.

**Methods:**

In this cross-sectional study, the levels of peripheral lymphocyte subsets were measured using flow cytometry in 53 patients with positive SARS-CoV-2 RT-PCR, for whom antibody testing of COVID-19 was performed.

**Results:**

The white blood cell, neutrophil, and lymphocyte counts consistently decreased in the IgM and IgG non-responder group, while the differences in the median value between the two study groups were found to be statistically significant only in terms of neutrophil counts (*P* = 0.024 for IgM response and *p-value =* 0.046 for IgG response, respectively). Moreover, the level of neutrophil-to-lymphocyte ratio was observed to be significantly lower in the IgM or IgG non-responder group compared to the IgM or IgG responder group (3.6 ± 3.1 vs. 6.3 ± 4.2; *p-value =* 0.021). The patients with IgM antibody response had a significantly lower CD20^+^ lymphocytes (11% versus 15% in the groups without IgM antibody response, *p-value =* 0.031), The percentages of NK cells and CD4^+^ T cells significantly increased in the patients with IgG antibody response compared to those without IgG antibody response (13% versus 10%, *p-value =* 0.028, and 41.5% versus 34%; *p-value =* 0.03, respectively). Moreover, the patients who produced IgM or IgG antibody had significantly higher percentages of total T lymphocytes (64% versus 54%; *p-value =* 0.017), CD4^+^ T cells (41% versus 34%; *p-value* = 0.038), and NK cells (13% versus 9%, *p-value* = 0.023) compared to the group with no serological response. No significant difference was observed in the percentage of other lymphocyte subsets, including CD8^+^ T cells, T_reg_ cells, and CD19^+^ B cells.

**Conclusion:**

Our results suggest that the total T cells, CD4^+^ T cells, and NK cells percentages are linked to serological response. Moreover, our findings suggested that neutrophil absolute counts and neutrophil-to-lymphocyte ratio may be valuable predictors of IgM or IgG antibody response.

**Supplementary Information:**

The online version contains supplementary material available at 10.1186/s12879-021-06278-2.

## Background

Coronavirus Disease 2019 (COVID-19), which was caused by severe acute respiratory syndrome coronavirus 2 (SARS-CoV-2), has emerged as an international concern. Although the number of reports on different aspects of COVID-19 is currently increasing, the manner of immune cell subsets changed during COVID-19 has remained principally unclear yet [1].

It was shown that there are four major types of structural proteins of coronavirus, including the spike surface glycoprotein (S), small envelope protein (E), matrix protein (M), and nucleocapsid protein (N) [2]. Several studies have previously investigated the antibody responses to N protein or S glycoprotein in COVID-19 patients [2–6]. As a result, it has been reported that the detection of antibodies against the N protein of SARS-CoV-2 is more sensitive than that of the S glycoprotein antibodies, particularly at the early stage of this infection [7].

Lymphocytes in peripheral blood are heterogeneous, differing by cellular surface molecules such as CD3^+^, CD4^+^, and CD8^+^ (T cells); CD19^+^ and CD20^+^ (B cells); and CD16^+^ CD56^+^ (NK) cells are involved in the humoral and cellular immunity against viral infections. Moreover, regulatory T (T_reg_) lymphocytes play a vital role in suppressing excessive immune responses to pathogens; however, the molecular mechanisms involved in the regulation of forkhead box P3 (FOXP3) expression and antigen-specific response of T_reg_ cells in COVID-19 are unclear yet [8].

There is a growing number of studies on evaluating lymphocyte subset counts such as CD4^+^ and CD8^+^ T cells, B cells, and NK cells, which are now as prognostic factors for COVID-19 disease’s severity [9]. However, the lymphocyte subsets analyses of both IgM and IgG responders and non-responders during the periods after the onset of symptom have not been conducted so far.

The changes in peripheral lymphocyte counts as well as the transition of lymphocyte subgroups may suggest some possible mechanisms in the pathogenesis of SARS-CoV-2 infection [8]. Thus, it is important to clarify the characteristics of lymphocyte subsets in COVID-19, because these could consequently provide novel insights to explore the immune mechanism. Although recent studies reported a clear decrease in peripheral lymphocytes in COVID-19 patients, any alteration in the subsets still is unknown [10, 11].

On the other hand, the detailed data on IgG and IgM responses in the COVID-19 patients are very poor. So, the investigation of different lymphocyte subsets that could trigger significant antibody responses in COVID-19 patients, is crucial. In this regard, this study aimed to evaluate immune cell profiling of COVID-19 patients with and without antibody responses. We performed our article in terms of the STROBE guideline (https://www.strobe-statement.org/index.php?id=available-checklists).

## Methods

### Study design, setting, and participants

This study was a cross-sectional study, and the required data were obtained from the Masih Daneshvari hospital, as the referral center of lung diseases, Tehran, Iran. A total of 53 admitted cases of COVID-19 were recruited between February 23 and March 26, 2020. Accordingly, COVID-19 in these cases was confirmed by performing a reverse transcription-polymerase chain reaction (RT-PCR) assay on oropharyngeal or nasopharyngeal samples. We only included those cases who were confirmed as COVID-19 and those who had both flow cytometry and SARS-COV2 IgM and IgG assays results at the same time. We excluded the suspected cases with negative results of RT-PCR.

All the hospitalized patients were symptomatic and we classified the confirmed COVID-19 patients into moderate, severe, and critical groups based on the severity of the disease [12]. Severe cases presented at least one of the following criteria: [1] respiratory distress (respiration rate ≥ 30 times/min) [2]; blood oxygen saturation (SpO_2_) ≤ 93%; and [3] arterial partial pressure of O_2_ to a fraction of inspired oxygen (PaO_2_/FiO_2_) ratio ≤ 300 mmHg. Notably, critical cases of respiratory failure required a mechanical ventilation and/or shock and those with multiple organ failure required intensive care unit therapy.

From each patient, two blood samples were taken, 2 ml for serological assay and 2 ml was put in a tube containing EDTA as anticoagulant for flow cytometry. The specimens were then transferred to a laboratory and tested by passing up to six hours from the sampling. Thereafter, both flow cytometry and SARS-COV2 IgM and IgG assays were performed at the same time.

### Enzyme-linked immunosorbent assay (ELISA)

In order to conduct a serologic study, serum was isolated from the obtained blood samples and then subjected to indirect ELISA. Accordingly, the ELISA methods used in this study were SARS-COV2 IgM and IgG assays by Pishtaz Teb Diagnostics (Iran) catalog no. PT-SARS-CoV-2.IgM-96 and catalog no. PT-SARS-CoV-2. IgG-96. The performed procedure was as follows:

ELISA was performed on the serums isolated from the blood samples. 96 micro-well plates coated by SARS-CoV-2 nucleocapsid antigen were used as solid phases. 100 μl of 1/100 diluted human serum in specific assay buffer was then added to each micro-well and incubated for about 30 min at room temperature. Thereafter, the wells were washed five times with 250 μl of wash buffer and allowed to dry. Afterward, 100 μl of anti-human antibody conjugated with horseradish peroxidase (HRP) was added to them and they were incubated for 30 min at room temperature before being washed with 250 μl of washing buffer for five times. Finally, 100 μl of TMB (Tetramethylbenzidine) chromogen-substrate was added to each micro-well and they were then incubated for 15 min before being read with 100 μl of the stop solution. IgG as mouse monoclonal anti-human heavy chain (γ) and IgM as mouse monoclonal anti-human heavy chain (μ) were HRP conjugated. In each run, negative and positive controls were used. The patients and the controls’ serums were tested twice and the mean of optical density (OD) was also calculated. In terms of the manufacturer’s recommendation, the cut off value for IgM calculation was OD of the negative control plus 0.20 and the cut off value for IgG calculation was OD of the negative control plus 0.15. For each sample, the cut off index (COI) was calculated by ratio. It is noteworthy that COI less than 0.9 was considered as negative, 0.9–1.1 was considered as borderline, and ≥ 1.1 was reported as positive.

The five highest positive serums obtained from the convalescent patients with a cut off of about 10 were serially diluted for control, all of which were positive until 1/10 dilutions. The ELISA reader was Stat Fax 4200 by AWARENESS TECHNOLOGY Company, which was calibrated by specific calibration plate and qualitatively controlled by Pishtaz Teb Elisa check kit. For both IgM and IgG detection, the manufacturers claimed sensitivities of 79.4 and 94.1% as well as specificities of 97.3 and 98.3%, respectively.

### Flow cytometry analysis

The evaluations of antibody response and lymphocyte subsets were performed in each patient in the same day. Percentage and counts of (cells/μL) CD3^+^, CD4^+^, and CD8^+^ T cells; CD19^+^ and CD20^+^ B cells; CD16^+^ CD56^+^ NK cells; and CD4^+^/CD25^+^/FOXP3^+^ regulatory T cells were also measured at this stage. Briefly, the samples were centrifuged and then red blood cells were removed by lysis buffer. White blood cells were harvested, and then washed with cold PBS. Afterward, the cell-surface Fc receptors were blocked by 2.4 G2 (PharMingen, San Diego, CA, USA). As well, phycoerythrin (PE)-conjugated anti human CD4^+^, CD19^+^, CD56^+^ antibodies (PharMingen) were used to stain CD4^+^ T cells, CD19^+^ B cells, and CD56^+^ NK cells. Moreover, anti -Human CD8 and CD16 allophycocyanin (APC) conjugated antibodies were used to stain CD8^+^ and CD16^+^ cells; and fluorescein sothiocyanate (FITC)-conjugated antibody (PharMingen) were applied for CD3^+^ T cells in terms of the manufacturer’s instructions. After the incubation for 4–30 min in a dark place, the cells were washed and 10,000 events were analyzed on a FACSCalibur™ flow cytometer (Becton Dickinson, San Jose, CA, USA). For this purpose, peripheral blood samples of the patients were firstly isolated by CD45 versus side scatter of lymphocyte population and then conjugated antibodies were analyzed as presented with two and three colors in the specified lymphocyte population. Notably, the dead cells were removed by staining with Propidium Iodide (PI). For the final analysis, FlowJo software version 8 was used.

### Statistical analysis

All the statistical analyses in this study were performed using SPSS version 20.0 software. As well, graphical presentations were conducted using GraphPad Prism version 7.0 (GraphPad Software, Inc., CA, USA).

In the current study, continuous variables were presented as median and interquartile range (IQR), and categorical variables were expressed as percentages in different categories. Means for the continuous variables were compared using independent group t-tests when the data were normally distributed; otherwise, the Mann-Whitney test was used. The Chi-squared test or Fisher’s exact test was applied for the category variables. A two-sided *p-value* < 0.05 was considered as statistically significant.

An unpaired t test was applied to ascertain significant differences in the lymphocyte subsets percentage among the patients with COVID-19 between those with and without antibody responses. The differences among the study groups were performed using one-way ANOVA with post hoc t test that corrected the use of the method of Bonferroni for the normally-distributed continuous variables. The neutrophil to lymphocyte ratio was also calculated by dividing absolute neutrophil count to absolute lymphocyte count.

Finally, Spearman’s correlation tests were performed to find a possible significant correlation between the level of peripheral lymphocyte subsets within the antibody responder group.

## Results

We analyzed the levels of lymphocyte subsets using flow cytometry in whole blood samples of the patients with positive SARS-CoV-2 RT-PCR, for whom antibody testing for COVID-19 was performed.

### Baseline characteristics of the patients

In the current study, 53 confirmed cases of COVID-19 were included. In all of them, the diagnosis of COVID-19 was confirmed by SARS-CoV-2 RT-PCR. Among them, the median age was 54 years old (IQR, 39–63), and 29 patients (55%) were women. Diabetes mellitus (30%) and hypertension (19%) were the most common comorbidities. Based on the severity of the disease, 19 patients (36%) showed a moderate form of the disease, while 21 patients (40%) and 13 patients (24%) were classified into severe and critical groups, respectively.

In blood tests, the levels of leukocytes, neutrophils, and lymphocytes were below the normal range in 6 (11%), 4 (7.5%), and 17 (32%) patients, and above the normal range in 14 (26%), 15 (28%), and 2 (4%) patients, respectively. Thereafter, we compared the results of the assessment of all the patients with COVID-19 with antibody response with those of the patients who did not develop any detectable IgG and/or response during their hospitalization period. Of note, the average time between the onset of symptoms and the serology test and antibody detection was 15 ± 6 days.

Among 53 patients include in this study, 39 (74%) and 40 (75.5%) cases produced anti-SARS-CoV-2 IgM and anti-SARS-CoV-2 IgG, respectively. Accordingly, the mean age of those who produced antibodies was lower than that of the COVID-19 patients with negative serology tests (51.1 ± 14.1 vs. 60.3 ± 19.3 years old, respectively; *p-value =* 0.08).

Ninety-five percent of the patients (*n* = 18) with moderate COVID-19 developed SARS-CoV-2-specific IgG antibodies in their serums, which were significantly higher than those of the participants of the groups with both severe (*n* = 16, 76%) and critical COVID-19 (*n* = 6, 46%; *p-value* = 0.007). Of the patients with moderate COVID-19, 15 cases (79%) developed SARS-CoV-2-specific IgM antibodies, while 17 patients and 7 patients with severe and critical COVID-19 developed the detectable IgM antibodies, respectively (*p-value* = 0.176). The levels of both IgM and IgG antibodies in serums of the patients with critical COVID-19 were found to be lower than those of the patients with moderate and severe diseases; however, these were not significant (1.3 (0.4–6.0), 6.1 (1.9–9.), and 6.4 (2.6–15.1) for IgM (*p-value* = 0.13); as well as 0.7 (0.4–14.2), 14.4 (8.8–14.9), and 14.6 (3.1–15.1) for IgG (*p-value* = 0.18), respectively).

**Fig. 1:**
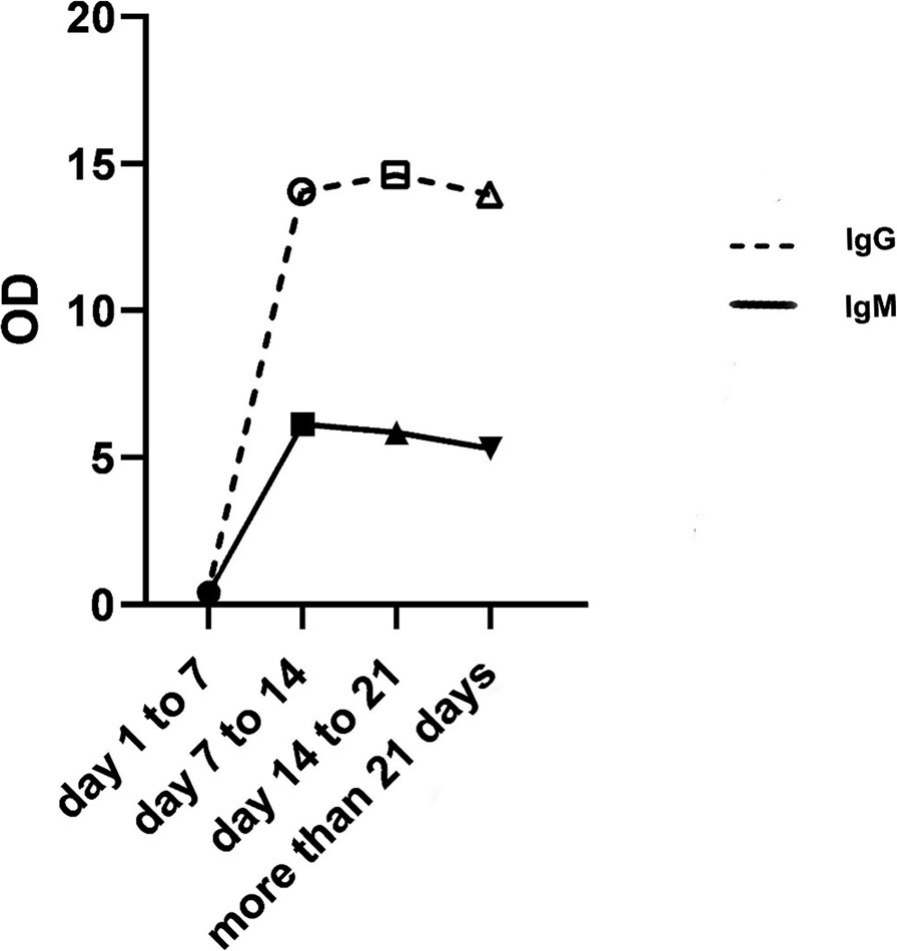
The mean titers of SARS-Co-V-2 nucleocapsid specific IgM and IgG antibodies responses in patients with COVID-19 during the time after symptom onset

The seropositive rates of IgM and IgG antibodies by passing one week from the onset of the symptoms were 14 and 29%, respectively. Additionally, the seropositive rates of IgM and IgG antibodies were 62.5 and 66% two weeks post symptoms’ onset; while during 3 weeks post symptoms’ onset, the seropositive rates of IgM and IgG antibodies reached 72 and 74%, respectively. Within 3 weeks post symptoms’ onset, the seropositive rates of either IgM or IgG antibody maintained at 86%. The results showed that by passing one week from the symptoms’ onset, the IgM, IgG antibody titers were low, but these significantly increased over time (*p-value =* 0.001 and *p-value =* 0.012. respectively) (Fig. 1).

The white blood cell, and neutrophil and lymphocyte counts consistently decreased in the IgM and IgG non-responder groups, while the differences in the median value between the two study groups was statistically significant only for neutrophil counts (*p-value* = 0.024 for IgM response and *p-value* = 0.046 for IgG response). As well, the neutrophil counts significantly decreased in the serological responder group compared to the IgM antibody or IgG antibody in the non-responder group (4411× 10^6^ cells/L [IQR: 2800–6862 × 10^6^ cells/L] and 7699.5 [IQR: 5704–10,354], respectively; *p-value* = 0.035). Moreover, the neutrophil-to-lymphocyte ratio was found to be significantly lower in the IgM or IgG non-responder group compared to the IgM or IgG responder group (3.6 ± 3.1 vs. 6.3 ± 4.2, respectively; *p-value* = 0.021).

The median levels of erythrocyte sedimentation rate and C-reactive protein were higher in the IgM and IgG responder groups than those of the IgM and IgG non-responder groups; however, these differences were not significant (*p-value* = 0.49 for both IgM and IgG responses).

Table 1 shows the median absolute values of lymphocyte subsets in the groups with and without positive results of COVID-19 serology tests.

**Table 1:** The characteristics and immunological profiles of the patients with and without antibody response

Variables	IgM antibody			IgG antibody		
	Non-responders (n = 14) Median (IQR)	Responders (*n* = 39) Median (IQR)	*P* value	Non-responders (n = 13) Median (IQR)	Responders (*n* = 40) Median (IQR)	*P* value
Age (year)	54 (33–68)	54 (39–60)	0. 82	59 (42–76)	53 (39–60)	0.94
Days after onset of symptom	9 (5–13)	14 (11–18)	**0.036**	10 (6–14)	14 (10–18)	0.23
Erythrocyte sedimentation rate (mm/h)	35 (25–72)	48 (35–69)	0.49	36 (26–73)	48 (34–69)	0.45
C-reactive protein (mg/L)	18 (14–40)	31.5 (19–42)	0.22	17 (7–34)	31.5 (19–41)	0.18
Interleukin 6 (pg/mL)	11.3 (8–15)	10 (6–16)	0.2	11 (8–15)	10 (6–16)	0.32
White blood cell count (× 10^6^ cells / L)	10,980 (6928–15,000)	7030 (5220–9980)	0.1	10,860 (7150–17,075)	7000 (5230–1024)	0.17
Neutrophil count (× 10^6^ cells / L)	6865 (5498–10,691)	4370 (2871–660)	**0.024**	7700 (5259–11,625)	4284 (2659–6840)	**0.046**
Lymphocyte count (× 10^6^ cells / L)	1878 (1160–2344)	1681 (1320–2000)	0.3	1776 (1042–2098)	1715 (1320–2131)	0.47
Total T cell counts (CD3^+^)	1148 (627–1465)	1003 (739–1370)	0.69	1019 (391–1305)	1100.5 (785–1412)	0.94
CD4^+^ T cell counts	685.5 (283–872)	663 (399–876)	0.82	580 (249–787)	693.5 (463–902)	0.23
CD8^+^ T cell counts	343 (216–449)	280 (180–420)	0.3	294 (117–451)	291 (193–417)	0.93
B cells (CD19^+^) counts	230 (125–418)	164.5 (105–237)	0.35	195.5 (107–303)	171.5 (110–243)	0.74
B cells (CD20+) counts	227.5 (126–398)	159.5 (104–234)	0.12	200 (109–288)	165 (109–242)	0.74
NK cell counts	136 (79–318)	151 (127–263)	0.95	135 (68–244)	158.5 (130–282)	0.69
Neutrophil to lymphocyte ratio	4.6 (2.2–9.6)	2.4 (1.2–4.1)	0.31	4.8 (2.5–9.9)	2.3 (1.3–4.1)	0.17

Data were presented as the median (interquartile range) and were analyzed using the Mann–Whitney test. IgM, immunoglobulin M; IgG, immunoglobulin G; NK, natural killer. *p value* < 0.05 was considered statistically significant

### Peripheral lymphocyte subsets in COVID-19 concerning IgM antibody response

**Fig. 2:**
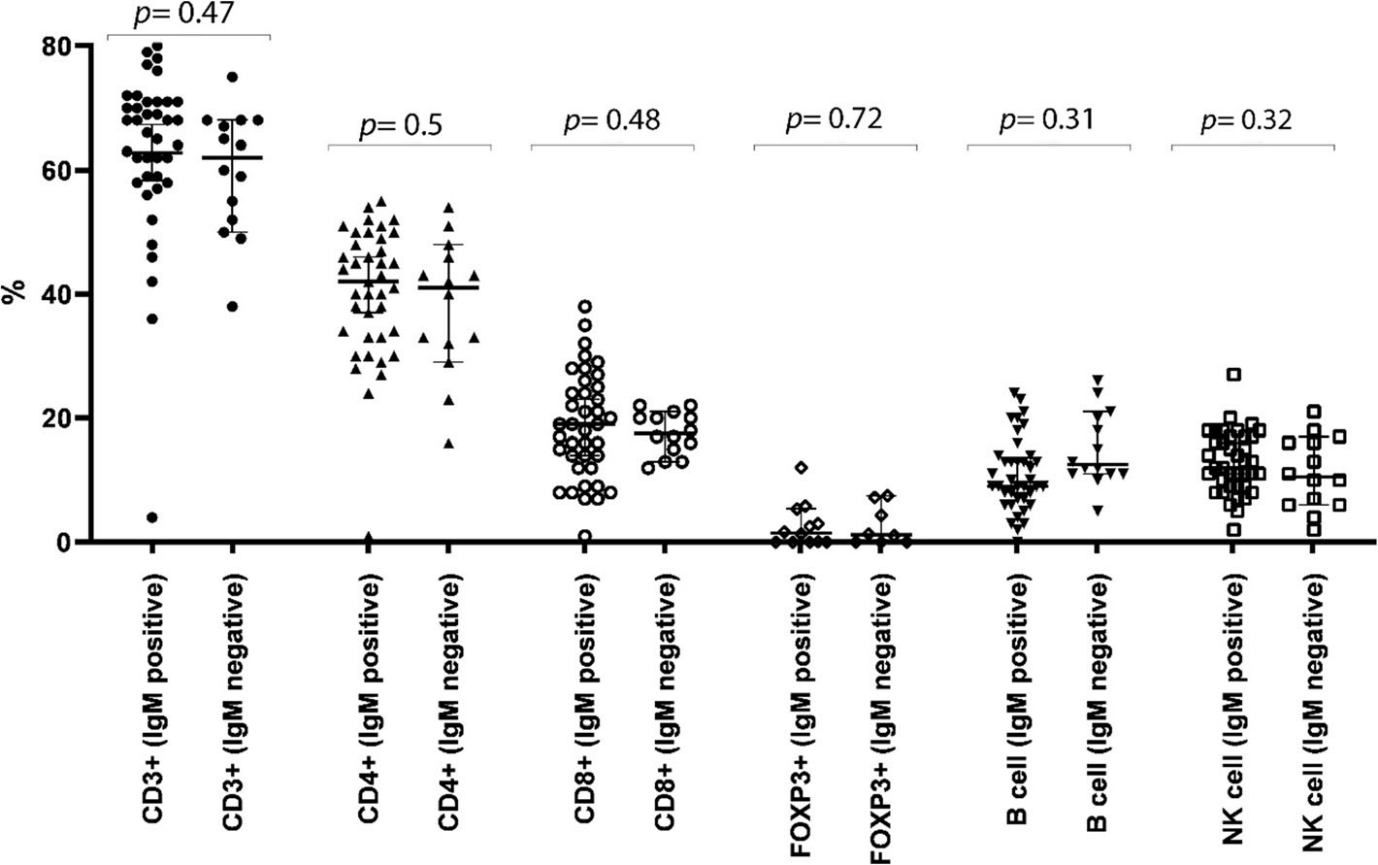
Titer comparison of the percentage of peripheral lymphocyte subsets in COVID-19 patients with and without IgM antibody response against SARS-Co-V-2 nucleocapsid

No significant difference was observed in the percentage of other lymphocyte subsets, including total T cells (*p-value* = 0.47), CD4^+^ T cells (*p-value* = 0.5), CD8^+^ T cells (*p-value* = 0.48), FOXP3^+^ T cells (*p-value =* 0.72), CD19^+^ lymphocytes (B cells) (*p-value =* 0.31), and NK cells (*p-value* = 0.32) between the groups with and without IgM antibody response (Fig. 2).

### Peripheral lymphocyte subsets in COVID-19 concerning IgG antibody response

**Fig. 3:**
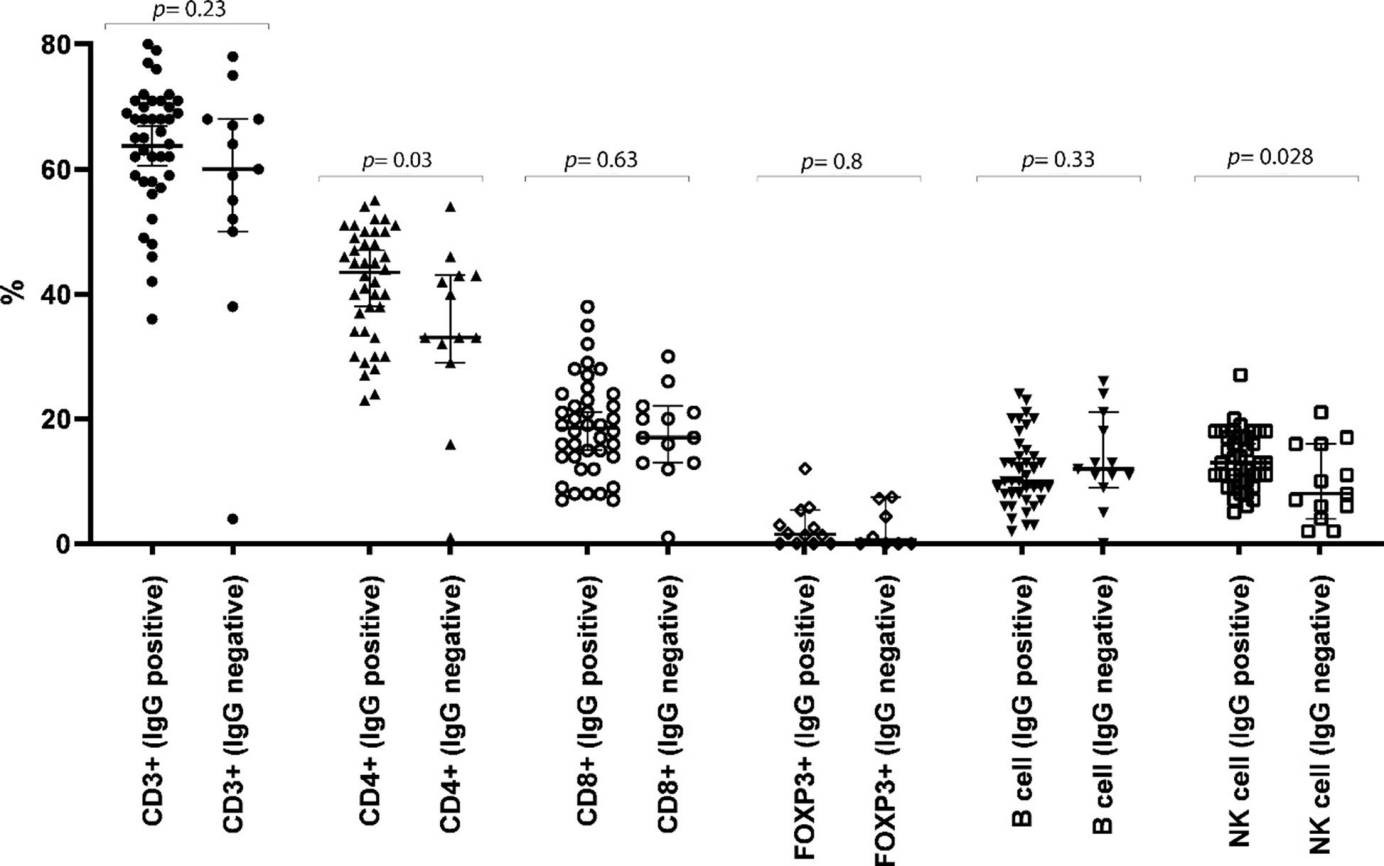
Titer comparison of the percentage of peripheral lymphocyte subsets in COVID-19 patients with and without IgG antibody response against SARS-Co-V-2 nucleocapsid

The percentages of NK cells and CD4^+^ T cells significantly increased in the patients with IgG antibody response compared to the group without IgG antibody response (13% versus 10%, *p-value =* 0.028; and 41.5% versus 34%, *p-value =* 0.03, respectively). However, no significant difference was observed in the percentage of the other lymphocyte subsets, including total T cells (*p-value* = 0.23), CD8^+^ T cells (*p-value* = 0.63), T_reg_ cells (*p-value* = 0.8), and CD19^+^ B cells (*p-value =* 0.33) (Fig. 3). Notably, the level of CD3^+^ T-cell was found to be correlated with NK cells within the positive IgG group (*r* = 0.446, *p-value* = 0.004), while CD4^+^ T-cell showed no significant correlation with NK cells (*r* = 0.310, *p-value* = 0.052).

### Peripheral lymphocyte subsets in COVID-19 concerning antibody response (IgM or IgG antibody)

**Fig. 4:**
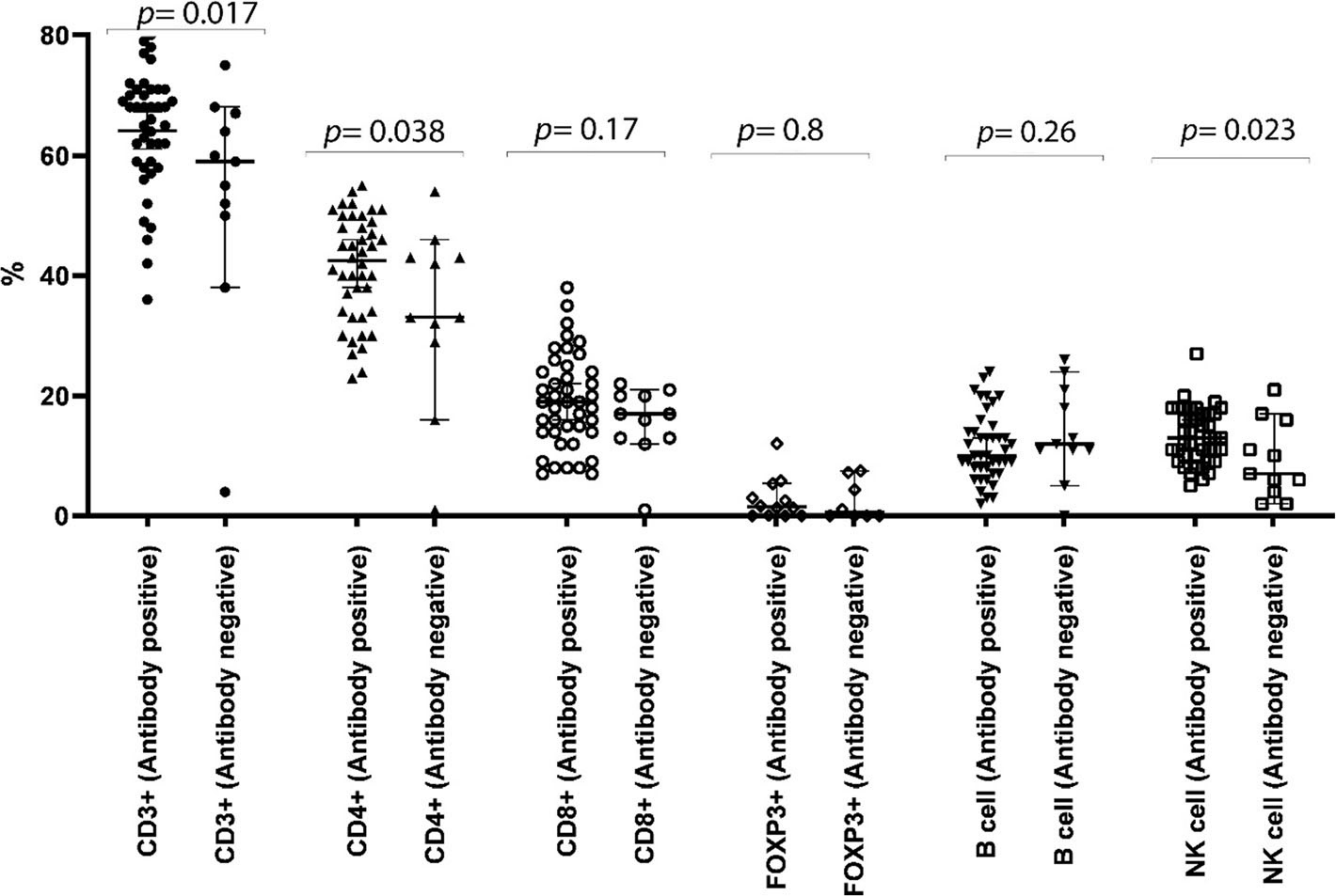
Titer comparison of the percentage of peripheral lymphocyte subsets in COVID-19 with serological response against SARS-Co-V-2 nucleocapsid

In comparison with the control group, the patients who produced IgM or IgG antibody had significantly higher percentages of total T lymphocytes (64% versus 54%; *p-value* = 0.017), CD4+ T cells (41% versus 34%; *p-value* = 0.038), and NK cells (13% versus 9%; *p-value* = 0.023). However, no significant difference was observed in the percentages of the other lymphocyte subsets, including CD8^+^ T cells (*p-value =* 0.17), T_reg_ cells (*p-value =* 0.8), and CD19^+^ B cells (*p-value =* 0.26) (Fig. 4). The level of CD3^+^ T cells and CD4^+^ T cells were also found to be correlated with NK cells within in all the cases of the responder group (*r* = 0436, *p-value* = 0.004 and *r* = 0.317, *p-value* = 0.041, respectively).

Examples of measuring the expression of the selected CD markers in the population of gated lymphocytes are shown in the supplementary data (Fig. S1).

### Lymphocyte subset levels and COVID-19 severity

Based on the severity of COVID-19, critical cases showed lower CD3^+^ T cells (*P* < 0.0001), CD4^+^ T cells (*p-value* = 0.001), CD19^+^ B cells (*P* = 0.025), and CD20^+^ B cells (*p-value* = 0.018). However, no significant difference was observed in CD8^+^ T cell (*p-value* = 0.91) and NK cell percentages (*p-value* = 0.078).

## Discussion

To the best of our knowledge, this was the first study focused on the phenotype of lymphocyte subsets in the COVID-19 patients with and without the production of IgM or IgG antibody against SARS-CoV-2 nucleoprotein. In the present study, the neutrophil counts significantly decreased in the serological responder group compared to both the IgM antibody and IgG antibody non-responder groups. Moreover, the level of neutrophil-to-lymphocyte ratio was found to be significantly lower in the IgM antibody and IgG antibody responder groups compared to the IgM antibody and IgG antibody non-responder groups (3.6 ± 3.1 vs. 6.3 ± 4.2, respectively, *p-value =* 0.021). Accordingly, this finding is important because the results of the recently performed studies identified the neutrophil-to-lymphocyte ratio as a powerful indicator of innate immunity that can provide a link between innate and specific immune responses [10, 13].

It is noteworthy that approximately 26% (*n* = 14) and 25% (*n* = 13) of the patients who were resulted as positive by RT-PCR, were found to be negative by performing the IgM or IgG antibody tests, and this is in consistent with the findings of the Guo et al.*’s* report [14]. According to the report by Gudbjartsson et al., no antibodies or undetectable levels of antibodies reactive to the S1 and N proteins were observed in some cases infected by SARS-CoV-2, even by passing 3 months from their infection [15].

Although some decreases were reported in CD4^+^ T cells, CD8^+^ T cells, B cells, and NK cells in the COVID-19 patients [1, 11, 16], knowledge on the role of lymphocyte subsets in humoral and cellular immune regulations in these patients is limited yet. In this study, we described the results of the serologic assays for the detection of antibodies to the N protein of SARS-CoV-2 and flow-cytometric analysis of lymphocyte subsets.

We found that the percentages of total T cells, CD4^+^ T cells, and NK cells had significantly greater reductions in the serological non-responder group compared to those who produced the IgM or IgG antibodies. This suggested that CD4^+^ T lymphocytes and NK cells play important roles in SARS-CoV-2 specific antibody response. Although the total number of NK cells has prominently decreased in patients with SARS-CoV-2 infection [8], the mean percentage of CD16^+^-CD56^+^ T cells was found to be significantly lower in the patients who did not produce IgM or IgG antibody. Furthermore, the significant difference between the groups with and without IgG antibody response in NK cells suggested its possible role in the regulation of IgG antibody production that is in line with the results of the study by Zheng et al. who reported higher numbers of NK cells in patients recovered from COVID-19 [17]. Moreover, NK cells might play important roles in SARS-CoV-2 clearance, T cell responses, and immunopathology of COVID-19 [18, 19].

A number of previous studies have reported the roles of T_reg_ cells in human immune-mediated diseases and immunological homeostasis [20–22]. However, in our study, the percentage and count of FOXP3^+^ T cells, as a T_reg_ cell–specific marker, did not differ among the COVID-19 patients with and without IgM or IgG antibody response.

Although humoral immune response may be correlated with protection [23], evaluation of neutralizing antibodies is more important. Since the detection of neutralizing antibodies was not a part of our study, so the neutralizing activities of the detected IgG antibodies remained unknown. Moreover, we only detected an antibody against the N protein of SARS-CoV-2; therefore, more studies should be conducted on the detection of antibody against the S protein along with the detailed analysis of immune cell compositions, in order to evaluate patient’s recovery stage comprehensively.

### Study strengths and limitations

The main strength of this study was the evaluation of immune cell profiling of the COVID-19 patients with and without IgM or IgG antibody production against SARS-CoV-2 nucleoprotein to demonstrate the possible role of lymphocyte subsets in humoral response. There are many unknowns in COVID-19 and there is limited data on the roles of lymphocyte subsets in both humoral and cellular immune regulations in patients with COVID-19. Significant reductions observed in the percentages of total T cells, CD4^+^ T cells, and NK cells suggested that these cells might play an important role in SARS-CoV-2 specific antibody response. Moreover, the neutrophil-to-lymphocyte ratio might be considered as a powerful indicator for SARS-CoV-2 immune response. However, the generalizability of our results to other settings may be limited due to the possible differences among health systems in different countries.

We acknowledge several limitations in this study. No follow-up evaluation of the patients to time to seroconversion was performed and we only compared their antibody responses during the time of their hospital stay. Moreover, the analysis of immune cells considering serological status was not stratified according to the severity of disease due to the small sample size of each study group. Finally, our study only included the hospitalized patients; therefore, mild symptomatic patients were missed.

## Conclusion

In conclusion, our results suggest that total T cells, CD4^+^ T cells, and NK cells percentages are linked to serological response. Moreover, our findings suggested that neutrophil absolute counts and neutrophil-to-lymphocyte ratio may be valuable predictors of IgM or IgG antibody response. Due to the emerging nature of COVID-19, our knowledge on its immunology still is limited. Therefore, further studies are necessary for better understanding the complex correlation between cellular and humoral immunities against SARS-CoV-2. These data could be helpful in vaccine design and targeted therapeutic options.

## Supplementary Information

**Additional file 1 Fig. S1.** Example of measuring the expression of selected CD markers in the population of gated lymphocytes

## Data Availability

The patient data and datasets used and/or analyzed during the current study are available from the corresponding author on reasonable request.

## Abbreviations

SARS-CoV-2: Severe acute respiratory syndrome coronavirus 2
COVID-19: Coronavirus Disease 2019
RT-PCR: Reverse transcription polymerase chain reaction
