# Supplementary material for: Immune cell profiling and antibody responses in patients with COVID-19
Source: BMC Infect Dis. 2021 Jul 5;21:646. doi: 10.1186/s12879-021-06278-2 (PMC8256640; doi:10.1186/s12879-021-06278-2)
Supplement: Supplementary file 1 — Additional file 1 Fig. S1. Example of measuring the expression of selected CD markers in the population of gated lymphocytes [file 12879_2021_6278_MOESM1_ESM.docx]

**Figure S1:** Example of measuring the expression of selected CD markers in the population of gated lymphocytes

| **%OF TOTAL** | **DIFFERENTIAL** |
| --- | --- |
| **10.0** | **Lymphocytes** |
| **12.0** | **Monocytes** |
| **78.0** | **Granulocytes** |

| **Reference** | **% of Lymphocyte** | **CD markers** |
| --- | --- | --- |
| **59-83** | **51.7** | **CD3^+^** |
| **31-59** | **41.3** | **CD4^+^** |
| **12-38** | **10.8** | **CD8^+^** |
| **3-22** | **22.0** | **CD16^+^** |
| **6-22** | **24.3** | **CD19^+^** |
| **6-22** | **24.1** | **CD20^+^** |
| **5-24** | **21.9** | **CD56+** |
|  | **10.2** | **CD3^+^CD8^+^** |
| **3-20** | **21.4** | **CD16^+^ CD56^+^** |

**
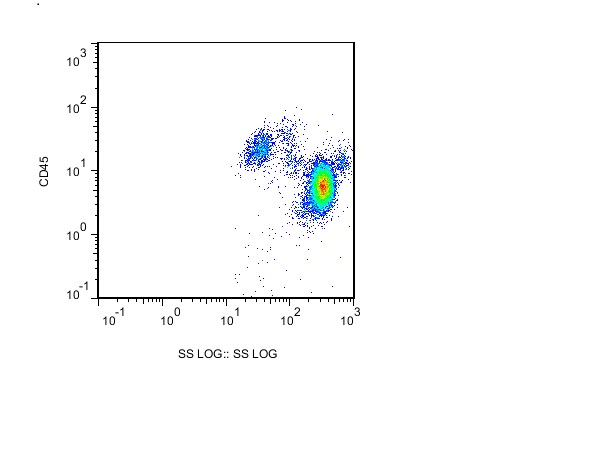
**

**
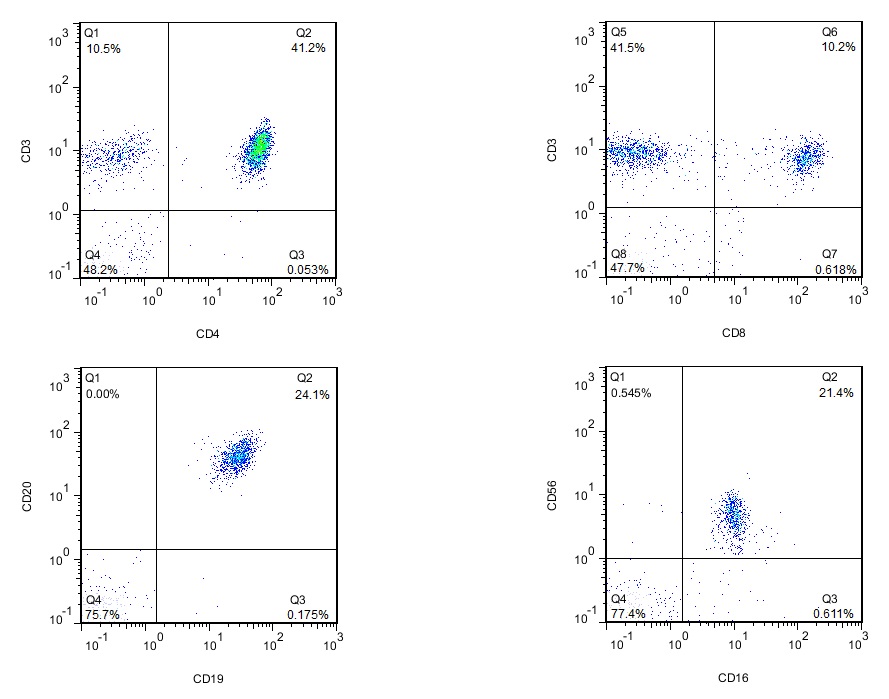
**

According to the figures, 10% lymphocytes were detected in the peripheral blood, with 41.3% CD4^+^, 10.8% CD8^+^, 21.4% CD16^+^56^+^, 24.3% CD19^+^, and 24.1% CD20^+^ expression in the gated lymphocyte population.
